# Carnitine Deficiency in Intensive Care Unit Patients Undergoing Continuous Renal Replacement Therapy

**DOI:** 10.31662/jmaj.2023-0200

**Published:** 2023-12-27

**Authors:** Kei Fukami, Kensei Taguchi

**Affiliations:** 1 Division of Nephrology, Department of Medicine, Kurume University School of Medicine, Kurume, Japan

**Keywords:** carnitine deficiency, CRRT, intensive care unit, dialysis

Carnitine, a relatively small molecule with 161 Da molecular weight, plays a critical role in accelerating fatty acid β-oxidation and subsequent energy production within the mitochondria. This natural substance is predominantly stored in the skeletal muscles and acts as a facilitator for transporting long-chain fatty acids into the mitochondria, initiating the cascade of β-oxidation and ATP synthesis. Consequently, long-term carnitine deficiency impairs the metabolism of long-chain fatty acids and energy production, disturbing cell viability and leading to general fatigue, muscle weakness, sarcopenia, left ventricular hypertrophy, and renal anemia. Although carnitine deficiency does not occur under normal physiological conditions and might be induced by special occasions such as performing dialysis, antibiotics usage, and genetic carnitine transporter abnormalities in mitochondria.

Herein, we reported a significant decrease in serum carnitine levels among patients undergoing hemodialysis (HD) caused by the systematic elimination of serum carnitine from the bloodstream during HD sessions, with approximately 70%–80% of circulating carnitine removed in a single session ^(1), (2)^. Despite compensatory replacement from skeletal muscle pools, prolonged exposure to HD treatment reportedly induces systemic carnitine deficiency, leading to heart failure, anemia due to hyporesponsiveness of erythropoietin stimulating agents, sarcopenia, and malnutrition in elderly patients with end-stage kidney disease. The risks of carnitine deficiency in HD patients have been underestimated in the clinical setting.

In our previous study, we explored the impact of carnitine deficiency and independent determinants of serum carnitine levels in patients with long-term HD ^(3)^. Significant correlations were found between LDL-cholesterol, serum creatinine, uric acid, Kt/V (an indicator of HD efficacy), and serum carnitine levels. Moreover, Kt/V was identified as an independent determinant of serum carnitine levels, suggesting that the high HD efficacy accelerates carnitine deficiency in malnourished elderly HD patients ^(3)^. Furthermore, skeletal muscle mass depletion, a common occurrence in long-term malnourished HD patients, accelerates carnitine deficiency. In addition, decreased appetite and/or restricted consumption of protein-rich foods reportedly lead to carnitine deficiency, hypocholesterolemia, and hypoalbuminemia in patients with HD. Therefore, the heightened HD efficiency with these characteristics poses a significant risk of carnitine deficiency. Moreover, continuous renal replacement therapy (CRRT) may contribute to carnitine deficiency in seriously ill patients in the intensive care unit (ICU) with conditions such as sepsis, acute kidney injury (AKI), and hepatitis.

CRRT, another blood purification modality, is employed in patients with severe conditions such as sepsis, AKI, and hepatitis in the ICU. CRRT is typically a safer treatment option than HD due to its lower required blood flow rate per unit time, resulting in minimal impact on hemodynamics. However, several concerns exist regarding the potential leakage of carnitine by CRRT, leading to severe carnitine deficiency.

The study conducted by Sgambat et al. focused on the association between CRRT and carnitine deficiency, particularly in children; their findings revealed that patients undergoing CRRT for an extended duration (≥3 weeks) were carnitine deficient, with those experiencing total carnitine and free carnitine deficiency facing significantly higher odds of death than normal levels ^(4)^. Considerably, Oi et al. investigated the prevalence of carnitine deficiency in ICU patients receiving CRRT and found that 52.3% of patients were in a state of carnitine deficiency ^(5)^. Furthermore, CRRT blood purification volume exhibited an inverse correlation with blood carnitine concentration, suggesting that higher dialysis volumes were linked to increased carnitine leakage, possibly leading to carnitine deficiency ^(5)^.

The difficulty in the management of CRRT lies in the delicate balance between effective blood purification and the potential for carnitine leakage. Reducing the blood purification volume possibly mitigates carnitine leakage; however, it decreases dialysis efficiency, making the uremic toxin removals challenging. Prolonged CRRT introduces the possibility of exacerbating carnitine deficiency, potentially manifesting symptoms such as fatigue, muscle weakness, heart failure, and anemia. Additionally, severe conditions like sepsis, AKI, hepatitis, and others are anticipated to decrease carnitine synthesis in the kidney and liver. The disruptions of the carnitine pool equilibrium due to sarcopenia in the ICU is also expected to intensify carnitine deficiency (Figure 1).

**Figure 1.**
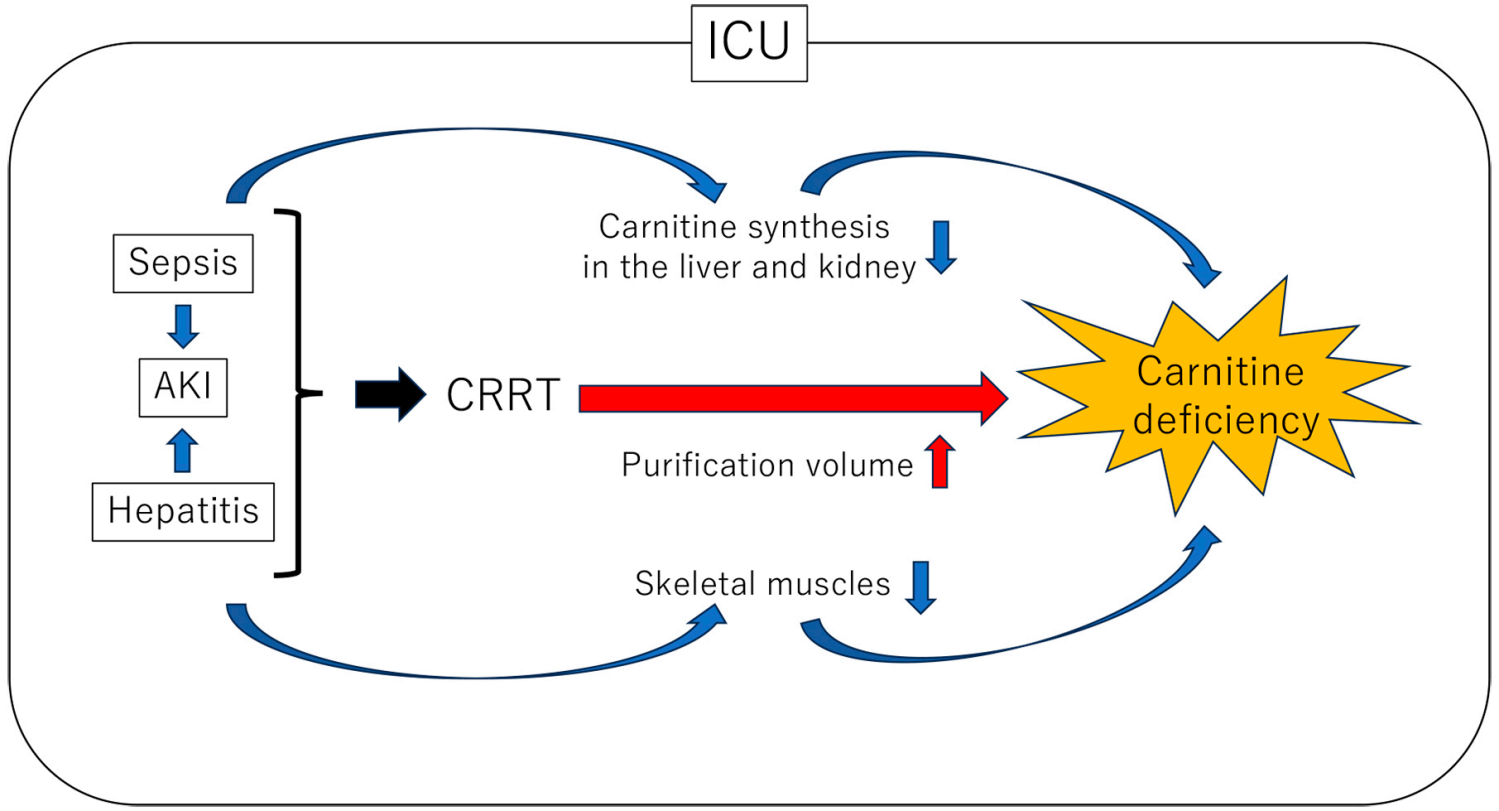
Carnitine deficiency determinants in severe ill conditions undergoing CRRT AKI: acute kidney injury; CRRT: continuous renal replacement therapy; ICU: intensive care unit.

Considerably, the L-carnitine supplementation during CRRT emerges as a promising therapeutic strategy for improving outcomes in patients with severe illness. Potential interventions against carnitine deficiency could be a crucial, multifaceted approach to optimize patient care and enhance the quality of life for critically ill patients undergoing CRRT in the ICU.

## Article Information

### Conflicts of Interest

KF received honoraria and lecture fees from Otsuka Pharmaceutical Co.
